# ^99m^Tc-MAG3 scintigraphy for the longitudinal follow-up of kidney function in a mouse model of renal ischemia-reperfusion injury

**DOI:** 10.1186/2191-219X-2-2

**Published:** 2012-01-20

**Authors:** Tanja Herrler, Hao Wang, Anne Tischer, Peter Bartenstein, Karl-Walter Jauch, Markus Guba, Markus Diemling, Cyril Nimmon, Marcus Hacker

**Affiliations:** 1 Department of Surgery, Campus Großhadern, University of Munich, Munich, 81377, Germany; 2 Department of Nuclear Medicine, University of Munich, Munich, 81377, Germany; 3 Hermes Medical Solutions, Skeppsbron 44, Stockholm, 111 30, Sweden; 4 Chiang Mai, Thailand

**Keywords:** fractional uptake rate, ischemia-reperfusion injury, ^99m^Tc-MAG3 scintigraphy, renal clearance, tubular function

## Abstract

**Background:**

Experimental models are essential tools in the development and evaluation of novel treatment options, but the preclinical model of renal ischemia-reperfusion injury is limited to the retrieval of (very) early functional data, leaving the pivotal long-term outcome unknown. The present study applies technetium-99m-mercapto-acetyl-tri-glycine [^99m^Tc-MAG3] scintigraphy for the longitudinal follow-up examination of long-term kidney function after renal ischemia-reperfusion injury.

**Methods:**

Unilateral warm ischemia was induced in scid beige mice by vascular clamping of the kidney hilum for 40 min. ^99m^Tc-MAG3 scintigraphy was performed prior to injury, 8 and 14 days post ischemia. The fractional uptake rate [FUR] was calculated from scintigraphy data as a measure of renal clearance.

**Results:**

FUR demonstrated a significant functional impairment of the ischemic kidney 8 and 14 days after injury (*P *< 0.05 vs. baseline), while contralateral non-ischemic kidneys showed no significant changes. In histological analysis, ischemic kidneys exhibited tubular dilatation and cytoplasmic degeneration as signs of hypoxia without any evidence for necrosis.

**Conclusions:**

FUR enables the detection of renal dysfunction and longitudinal long-term follow-up examination in the same individual. Our model may facilitate preclinical therapy evaluation for the identification of effective renoprotective therapies.

## Background

Following kidney transplantation, renal ischemia-reperfusion injury contributes to allograft dysfunction and loss. Promising experimental results have failed to translate into therapies for clinical use. This may be partly explained by methodological limitations in the experimental setting. In the preclinical model of renal ischemia-reperfusion injury, functional data based on standard biochemical parameters can only be obtained in the acute phase of injury [1,2]. Specifically, serum creatinine levels were shown to represent a valid indicator of renal dysfunction only during the early post-ischemic period of 24 to 72 h [3].

Dynamic scintigraphy using the radioactive compound technetium-99m-mercapto-acetyl-tri-glycine [^99m^Tc-MAG3] was recently introduced as a technique for the determination of early functional changes in a murine model of renal ischemia-reperfusion injury [4]. The aim of the present study was to apply ^99m^Tc-MAG3 imaging for longitudinal follow-up studies of early and, most importantly, long-term kidney function after injury. The fractional uptake rate [FUR] was calculated in analogy to studies in humans [5,6] as a measure of renal clearance using a modified protocol and enabled us to collect valuable information on individual long-term changes. To demonstrate the high accuracy and sensitivity of ^99m^Tc-MAG3 scintigraphy-based FUR, the study was performed using severe ischemia in a mouse strain with combined immunodeficiency of B cells, T cells, and natural killer cells that is known to confer protection from ischemia-reperfusion injury based on current functional parameters [7].

## Methods

### Animals

Male severe combined immunodeficient [scid] beige mice, aged 10 weeks and weighing 22 to 24 g, were purchased from Charles River Laboratories (Sulzfeld, Germany). The animals were fed a standard diet and were allowed free access to water. All animal experiments were conducted in accordance with the institutional guidelines and were approved by the Administrative Panel on Laboratory Animal Care (Government of Upper Bavaria, Germany).

### Renal ischemia-reperfusion injury model

Mice were anesthetized by intraperitoneal injection with a combination of 0.05 mg/kg fentanyl, 0.5 mg/kg medetomidine, 5 mg/kg midazolam and were placed on a heated surgical pad to keep a constant body temperature. The right kidney was exposed through a median abdominal incision, and the mice were subjected to ischemia by clamping the renal pedicle with a nontraumatic microaneurysm clamp (B. Braun Melsungen AG, Melsungen, Germany) which was removed after 40 min. The incision was closed with a 5-0 suture and surgical staples. Postoperatively, anesthesia was antagonized by intraperitoneal injection of a combination of 2.5 mg/kg atipamezole, 0.5 mg/kg flumazenil, and 1.2 mg/kg naloxone.

### Renal scintigraphy

^99m^Tc-MAG3 (Technescan MAG3, Covidien, Neustadt/Donau, Germany) imaging was performed in analogy to a previously described protocol [4]. Briefly, after hydration with sterile saline and induction of anesthesia with a combination of 0.05 mg/kg fentanyl, 0.5 mg/kg medetomidine, and 5 mg/kg midazolam, the mice underwent whole-body scintigraphy using one detector of a triple-headed gamma camera (Philips - formerly Picker - Prism 3000XP, Cleveland, OH, USA) equipped with a low-energy high-resolution [LEHR] collimator. A LEHR collimator was favored over a pinhole collimator [PC] as PC provides higher resolution but at the cost of lower sensitivity. PC would be ideal for static imaging where the frame time could be lengthened. However, this study was a dynamic study with a frame time of only 5 s. The calculation of FUR utilizes the data pre-peak of the renogram, which usually occurs within the first 2 min of the study. High sensitivity in detection as well as resolutions is required to achieve a better signal to noise ratios.

Acquisition of a dynamic planar imaging study was started with the i.v. injection of a standardized dose of approximately 37 MBq of ^99m^Tc-MAG3. One frame per 5 s was collected with a total scan time amounting to 10 min. The image acquisition magnification was set to ×4; after that, the scan anesthesia was antagonized as described above. To determine the baseline renal function, ^99m^Tc-MAG3 imaging was carried out 4 days before the ischemia-reperfusion surgery. Postoperative renal scans were performed on days 8 and 14 for the assessment of early and long-term kidney functions.

### Image analysis

**Figure 1:**
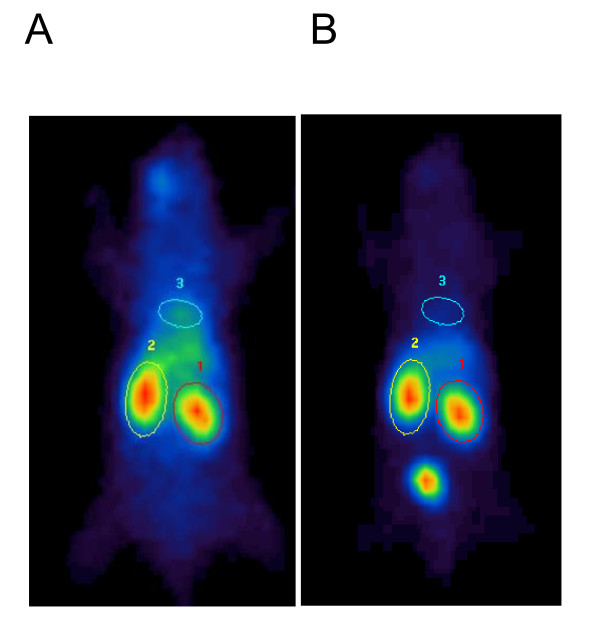
**Image files**. (**A**) Data summed between 30 and 90 s post injection. (**B**) Data summed between 5 and 6 min post injection. The standard manual ROI determination was performed for the left (ROI 1) and right kidneys (ROI 2), the cardiac region including the left ventricle (ROI 3), as well as subrenal kidney backgrounds (not shown).

Image files were analyzed using Hermes Dynamic Study Display software V4.0 (Hermes Gold V2.10, Hermes Medical Solutions, Stockholm, Sweden/London, UK) by standard manual region of interest [ROI] analysis of the whole body, including both kidneys and their background regions, as well as the site of injection, bladder, and blood pool over the heart region (Figure 1). The ROI that was defined over the heart region was used to generate a cardiac activity time curve. As a first approximation, this curve was considered to be proportional to a plasma clearance curve, *P*(*t*). Any extravascular component in the cardiac curve has been neglected as it is thought likely to be small within the first 2 min. Data were exported to Microsoft Excel to assess renal function. Renogram values represented as percentage of injected dose [%ID] were obtained by the division of the background corrected kidney ROI by the injection site corrected whole body ROI.

### Calculation of the fractional uptake rate

The FUR expresses the fraction of tracer in the blood taken up by the kidney per time unit as a measure of renal clearance. For the calculation of FUR, both kidney and cardiac curves were expressed in units of counts per second. The calculation was based on the equation previously proposed for patient studies [5], namely FUR *= P*(0) × (*k*_l _+ *k*_r_)/ID, where *k*_l _and *k*_r _(per second) are the slopes of the linear uptake [LU] segment of the Patlak-Rutland [PR] plots for the left and right kidneys, respectively. *P*(0) (counts per second) is the value of the plasma clearance curve *P*(*t*) at time zero, and it can be obtained by back extrapolation using a mono-exponential fit to *P*(*t*). The range used for this exponential fit was chosen to be identical with the combined extent of the individually determined LU segments. In an independent study [8], this choice of fitting range for the mono-exponential method has been shown to yield optimal results. The injected dose [ID] (counts per second) was estimated from the whole body count rate averaged over the first minute of the study and corrected for any residual activity in the region of the injection site. The detection sensitivity of activity from both the kidneys and the injected dose were considered equal, and no correction for renal depth was applied. This approximation was felt to be justified on account of the small scale of the animal model and the consistency of both size and anatomy within the sample population studied. The calculation of FUR was performed with the aid of a user-written renal curve analysis software program (Renalcurve V3.0B1) made available by Hermes Medical Solutions (Stockholm, Sweden/London, UK). A feature of this program is that the theoretical relationship between the linear segment of the PR plot and the plateau level of the renal impulse retention function [IRF] is utilized to identify the LU segments. The IRF is obtained by deconvolution analysis using a constrained least square method. In order to improve the robustness of the linear fit to the LU segments, an estimate of the expected intercept is calculated from the IRF and included in the fitting process as an additional data point. The resultant values for FUR were expressed in units of percent injected dose per minute. In order to test for possible dependence of the FUR values on the ROI size or position, the image data from a subset of eight kidneys were re-analyzed independently.

### Histological analysis

Tissue specimens, i.e., ischemia-reperfusion-injured right kidneys and contralateral non-ischemic kidneys, were collected for histological analysis. After paraffin embedding, histological sections were stained with hematoxylin and eosin. Structural damage was assessed by histological analysis.

### Statistical analysis

Statistical analysis was performed using paired or unpaired *t *test (two-sided). Our primary hypothesis was that ischemia-reperfusion decreases the kidney function measured by FUR. To test this hypothesis, FUR values were compared between post-ischemic and non-ischemic kidneys by paired *t *test. Data are expressed as mean ± SEM. *P *values < 0.05 were considered statistically significant.

## Results

### Renogram characteristics

**Figure 2:**
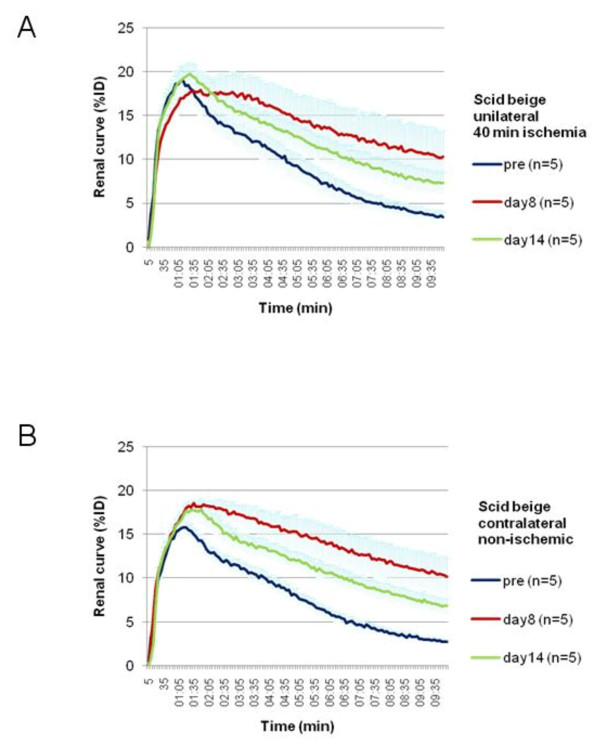
**Ischemic and contralateral non-ischemic kidney renograms before injury and at follow-up examinations days after ischemia**. (**A**) Renal ischemia of 40 min was associated with kidney dysfunction 8 days after injury reflected by a reduced slope and decreased uptake and persisted at day 14. (**B**) While the peak height increases after injury, no changes of the slope were found during follow-up which was 8 days after injury.

Renograms of ischemic kidneys exhibited functional loss in terms of flattening of the uptake slope, delayed peak, and reduced maximum height of the renogram 8 days following ischemia as compared with the initial measurement before ischemia. While the time to peak significantly improved, some flattening of the slope was still apparent by day 14 (Figure 2A). In comparison, renograms derived from contralateral non-ischemic kidneys showed only minor delay in time to peak and no changes in the uptake slope values (Figure 2B). The characteristics of a unilaterally delayed time to peak (due to prolongation of renal transit time) and reduced uptake function are similar to those commonly observed in patient studies in unilateral ischemia.

### Linear uptake segment

**Figure 3:**
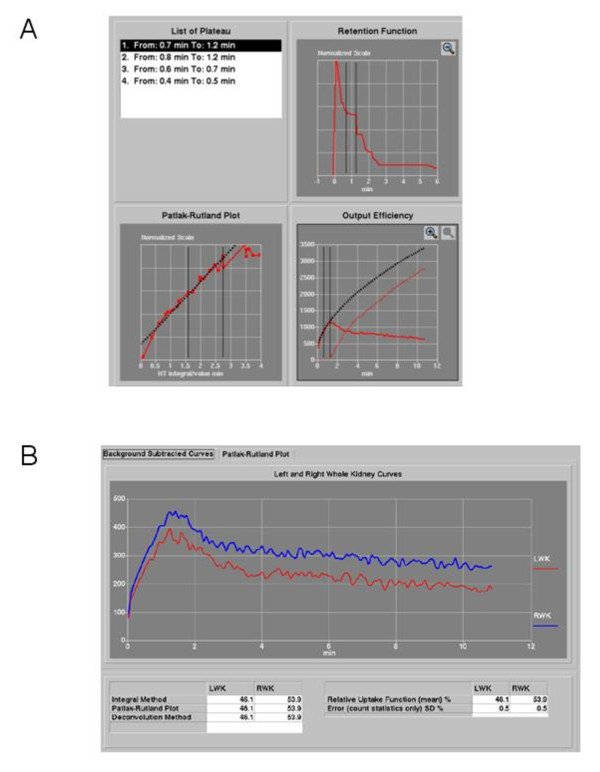
**Calculation of FUR**. (**A**) Example of the LU segment in the Patlak-Rutland plot. (**B**) DRF calculated as the percentage contribution to total uptake from the left and right kidneys.

Figure 3A illustrates an example of the LU segment in the PR plot (shown between vertical black lines) which has been automatically identified from the dominant plateau in the IRF occurring prior to the peak in the renogram [9]. This same 'uptake interval' is also shown on a graph of the integrated renal input curve fitted to the uptake phase of the background-corrected renogram curve. This latter graph may be used to calculate the renal output efficiency which was not used as a parameter in the present study. The triple graph display served as a quality control measure. In cases where the automatic detection of an appropriate LU segment was visually unsatisfactory, a manual adjustment was made with the aid of this display.

### Differential renal function

The tables in Figure 3B illustrate the differential renal uptake function [DRF] which is calculated as the percentage contribution to total uptake from the left and right kidneys. Values for DRF were calculated over the uptake interval by three different methods, namely (a) from the integral of the background subtracted renograms, (b) from the slopes of the PR plots, and (c) from the heights of the plateau in the IRFs. A close agreement between the values obtained by these three methods served as an additional quality control measure. Values for FUR_L _and FUR_R_, corresponding to the left and right kidneys, were then calculated by multiplication of the total FUR by DRF_L_/100 and DRF_R_/100, respectively. Repeat FUR values were obtained as a quality control check. From the subset of eight kidneys, the repeat FUR values (range, 0.9 to 23.4%ID/min) showed a linear correlation with a linear correlation coefficient *r *= 0.99, mean difference 0.65 ± 1.2%ID/min and a non-significant paired *t *test (*P *> 0.10).

### FUR for the determination of renal dysfunction following ischemia

**Figure 4:**
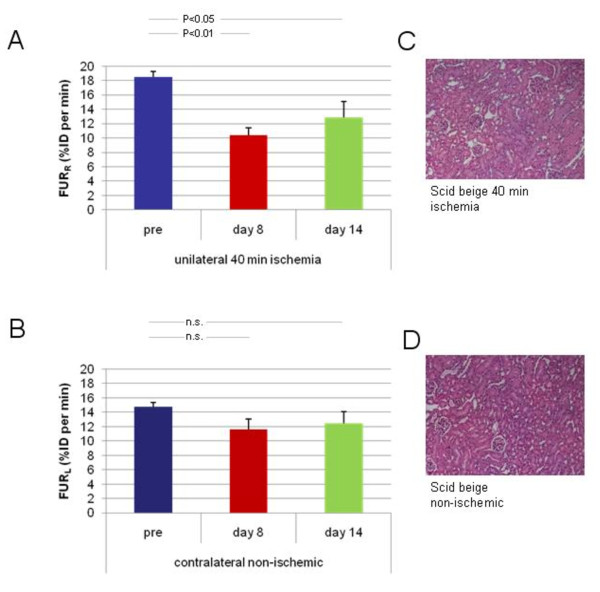
**FUR in ischemic and non-ischemic kidneys before and following unilateral warm ischemia of 40 min**. It is presented with corresponding histological sections. (**A**) Compared with the baseline, FUR calculation detected a significant decrease in kidney function both 8 and 14 days following severe ischemia. (**B**) Contralateral non-ischemic kidneys showed no significant changes in FUR after injury. (**C**) Ischemic kidneys exhibited structural changes as signs of hypoxia without any evidence for necrosis and inflammatory infiltration, respectively. (**D**) In the contralateral non-ischemic kidneys, no structural damage was found.

Unilateral ischemia of 40 min led to a significant reduction of FUR to 56.6 ± 7.0% (*P *< 0.01) 8 days post ischemia as compared with the baseline (Table 1, Figure 4A). While the mean FUR improved during follow-up, it remained significantly decreased even 14 days after injury (68.9 ± 11.2%; *P *< 0.05). Compared to the baseline, contralateral non-ischemic kidneys (Figure 4B) exhibited no significant changes in subsequent scintigraphy examinations neither by day 8 (78.7 ± 9.0%; *P *= n.s.) nor by day 14 (83 ± 8.3%; *P *= n.s.).

**Table 1:** Absolute FUR values and percentage of baseline values normalized to 100%

	Total (%ID/min)	FUR_L _(non-ischemic kidney)		FUR_R _(40 min ischemia)		*P*
		(%ID/min)	(% of baseline)	(%ID/min)	(% of baseline)	
Pre (baseline)	33.2	14.7 ± 0.6	100	18.5 ± 0.7	100	< 0.01
Day 8	21.9	11.6 ± 1.4	79	10.3 ± 1.1	56	n.s.
Day 14	25.3	12.5 ± 1.6	86	12.8 ± 2.2	69	n.s.

Absolute FUR values are presented as total (FUR_L _+ FUR_R_) as well as separated into the left non-ischemic (FUR_L_) and the right ischemic (FUR_R_) kidneys prior to ischemia (baseline), on days 8 and 14. The changes on days 8 and 14 are depicted as percentage of baseline values normalized to 100%.

### Histological analysis

Ischemic kidneys of scid beige mice exhibited tubular dilatation and cytoplasmic degeneration as signs of hypoxia. Tissue viability was excellent with no signs of necrosis. In addition, no inflammatory infiltration was found (Figure 4C). Contralateral non-ischemic kidneys showed no signs of structural damage (Figure 4D).

## Discussion

A reproducible and functionally relevant experimental setup for preclinical therapy evaluation enhances the identification of effective treatment strategies and the successful translation into clinical practice. However, the preclinical model of renal ischemia-reperfusion injury exhibits only limited validity because standard parameters of kidney function were shown to drop to baseline levels within a few days after nonlethal bilateral ischemia-reperfusion injury [3]. Most importantly, long-term effects as a result of the regenerative capability of the kidney and the efficacy of new therapeutic strategies remain unknown. Serum creatinine as the current gold standard indicator for renal dysfunction is mainly eliminated via glomerular filtration, but glomeruli are less susceptible to an event of ischemia-reperfusion, and tubular injury occurs before a decline in glomerular filtration is noted [10]. Thus, the function of the tubules representing the most critically affected structures is not adequately reflected by these standard methods.

To facilitate the determination of pivotal functional long-term outcome, we aimed to overcome these limitations. The calculation of FUR allowed for the reproducible assessment of renal dysfunction following ischemic injury during long-term follow-up. In particular, using FUR, we were able to detect renal dysfunction in immunodeficient mice known to be virtually protected from ischemia-reperfusion injury. In the present murine model using male mice prior to surgery (baseline), the right kidney exhibited significantly higher absolute FUR values than the left kidney. In humans, likewise, a significantly higher mean split function of the right kidney among males was described [11]. To our knowledge, this is the first study to report this unequal distribution of right and left renal function in the murine setting so far. As a consequence, the extent of renal dysfunction following injury is rather dependent on the initial baseline function depicted as the percentage of baseline values normalized to 100% on days 8 and 14 (Table 1). In reference to the baseline levels, a considerable functional decrease in the ischemic kidney was seen by day 8, although absolute values of FUR in the right ischemic kidney (FUR_R_) and the left non-ischemic kidney (FUR_L_) were quite similar during follow-up.

DRF allows for the determination of changes in relative renal function in reference to the contralateral kidney. In this particular setting, DRF may therefore not sufficiently depict but overestimate the functional effects of renal injury, as seen from the percentage baseline values normalized to 100% (Table 2). Moreover, changes in circulation dynamics are known to cause damage of non-injured nephrons due to hyperperfusion/hyperfiltration [12,13]. Transient dysfunction of the contralateral kidney has also been observed in some patient studies involving unilateral renal trauma [14]. In that paper, a hypothesis of autonomic reflex was suggested as a mechanism leading to a prolongation of parenchymal renal transit time and a flattening of the downslope in the renogram obtained for the untreated side. In the present model, potential damage of the contralateral non-ischemic kidney following unilateral renal ischemia-reperfusion injury must be assumed. This is supported by the transient flattening of the slope in the descending part of the renogram in the non-affected kidney on day 8, indicating renal dysfunction. On day 14, the slope becomes slightly steeper. Overall, no significant alterations in FUR are seen at the baseline, on day 8, and on day 14.

**Table 2:** DRF and percentage of baseline values normalized to 100%

	DRF_L _(non-ischemic kidney)		DRF_R _(40 min ischemia)		*P*
	(%)	(% of baseline)	(%)	(% of baseline)	
Pre (baseline)	44.3 ± 0.8	100	55.7 ± 0.8	100	< 0.01
Day 8	52.6 ± 1.2	119	47.4 ± 1.2	85	< 0.05
Day 14	49.9 ± 1.6	113	50.1 ± 1.6	90	n.s.

DRF is presented for the left non-ischemic (DRF_L_) and the right ischemic (DRF_R_) kidneys prior to ischemia (baseline), on days 8 and 14. The changes on days 8 and 14 are depicted as percentage of baseline values normalized to 100%.

A significant difference is seen between the DRF of the right injured and the left non-ischemic kidneys on day 8 (Figure 2B), while for FUR, this is not the case. Considering renal dysfunction of the contralateral non-ischemic kidney, as seen from the flattening of the slope of the descending part of the renogram, FUR would be expected to decrease as seen in Figure 1. In contrast, DRF of the non-ischemic kidney increases on day 8, as compared with the baseline. This may be misleading as relative improvement of the renal function is not necessarily associated with increased absolute function. DRF provides only relative values depending on both kidneys that may lead to inadequate conclusions on individual renal function. In contrast, the determination of kidney function using FUR in longitudinal studies bears the advantage of comparison with the initial baseline data of the healthy kidney.

Despite the great advantages of the present protocol, there may be some technical limitations. The study lacks the correction for any possible difference in depths of the left and right kidneys below the skin surface. Any such difference in depth would lead to some asymmetry in the value of DRF (and FUR) to some degree. Assuming a value of 0.12 for the linear attenuation coefficient for ^99m^Tc, one may estimate that a depth difference of 1.0 cm would result in a DRF ratio of 53%:47% (corresponding to a true ratio of 50%:50%). However, to obtain the observed distribution of 55.7%:44.3%, it would necessitate a depth difference of nearly 2.0 cm between the two kidneys which would seem unlikely for the small animal model.

FUR not only offers the advantage of expressing organ function in reference to tracer kinetics, but it has also been shown to be independent of patient size, gender, gamma camera, as well as time point and duration of examination [5]. Given the various interindividual differences in mice, this proves to be useful in the murine model as well. Our overall experience from the present study is that when provided care and attention to detail is paid to the implementation, then FUR seems to be a potentially very useful parameter. Importantly, this study demonstrates that a conventional gamma camera is sufficient enough to achieve highly reliable functional data.

In the present experimental setup, we investigated the functional outcome following a severe ischemia time of 40 min. In scid beige mice, this extent of injury resulted in a comparatively moderate reduction of renal function. In immunocompetent wild-type mice, a much more pronounced decrease in kidney function would be expected with a histologically distinct post-ischemic inflammation [15]. Histological findings in kidneys of scid beige mice were in accordance with the functional data. Tubules of ischemic kidneys only exhibited mild structural changes as signs of hypoxia without any evidence for severe damage. The absence of inflammatory infiltration in our model represents a feature of immunodeficient mice, supporting the notion of a severe ischemia-little injury model, and thus, the high reliability and sensitivity of the presented technique.

There are other important advantages of the utilized technologies. The assessment of standard renal function parameters requires kidney injury models using bilateral ischemia and unilateral ischemia with contralateral nephrectomy, respectively. The present technique enables the measure of relative renal function by the collection of separate data sets for the individual kidney so that injury may be limited to unilateral renal ischemia. Compensation of kidney function by the contralateral healthy kidney reduces morbidity and mortality while facilitating the use of severe injury models. It is important to note that ischemia times required for the desired tissue damage may vary depending on the utilized mouse strain. The clinical situation, of course, is often much more complicated. This type of acute unilateral kidney injury does not intend to represent a clinically realistic model. It rather serves as a simplified but useful model for future therapy evaluation in the field of kidney regeneration.

## Conclusion

Bearing in mind the poor prognosis of patients with renal failure and the shortage of transplants, our data could be of considerable importance for the identification of novel treatment modalities to improve kidney allograft function and survival. Before any new treatment modality should be translated into a clinical setting, reliable preclinical studies are absolutely mandatory. Our model may aid to develop the respective studies and to reproducibly demonstrate the functional relevance of the investigated treatment regimen for renal and vascular regeneration.

## Competing interests

The authors declare that they have no competing interests.

## Authors' contributions

TH contributed in the concept and design of the study as well as in the analysis and interpretation of data. HW participated in the analysis of data, and AT carried out animal handling and analysis of data. PB and K-WJ both revised the manuscript critically for important intellectual content. MG participated in the interpretation of data and in the revision of the manuscript critically. MD participated in the analysis and interpretation of data and in revising the manuscript critically. CN provided consultative advice on the calculation of FUR and independently re-analyzed a small subset of data as a quality control check. MH contributed in the concept and design of the study as well as in the analysis and interpretation of data, in revising the manuscript critically for important intellectual content, and in the final approval of the manuscript. All authors read and approved the final manuscript.

## References

[B1] Palm M Lundblad A Creatinine concentration in plasma from dog, rat, and mouse: a comparison of 3 different methods Vet Clin Pathol 2005 34 232 236 10.1111/j.1939-165X.2005.tb00046.x 16134070

[B2] Meyer MH Meyer RA Jr Gray RW Irwin RL Picric acid methods greatly overestimate serum creatinine in mice: more accurate results with high-performance liquid chromatography Anal Biochem 1985 144 285 290 10.1016/0003-2697(85)90118-6 3985323

[B3] O'Donnell MP Burne M Daniels F Rabb H Utility and limitations of serum creatinine as a measure of renal function in experimental renal ischemia-reperfusion injury Transplantation 2002 73 1841 1844 10.1097/00007890-200206150-00025 12085012

[B4] Roberts J Chen B Curtis LM Agarwal A Sanders PW Zinn KR Detection of early changes in renal function using 99mTc-MAG3 imaging in a murine model of ischemia-reperfusion injury Am J Physiol Renal Physiol 2007 293 F1408 F1412 10.1152/ajprenal.00083.2007 17634403 PMC3373432

[B5] Rutland M Que L Hassan IM "FUR"--one size suits all Eur J Nucl Med 2000 27 1708 1713 10.1007/s002590000337 11105828

[B6] Šámal M The 14th international symposium on radionuclides in nephrourology Semin Nucl Med 2011 41 3 10 10.1053/j.semnuclmed.2010.09.004 21111855

[B7] Zhang ZX Wang S Huang X Min WP Sun H Liu W Garcia B Jevnikar AM NK cells induce apoptosis in tubular epithelial cells and contribute to renal ischemia-reperfusion injury J Immunol 2008 181 7489 7498 19017938 10.4049/jimmunol.181.11.7489

[B8] Kubinyi J Nimmon C Samal M Kotalova D Smidt V Brabec V Bergmann H Improved gamma-camera method to calculate renal clearance using fractional renal uptake (FUR) Eur J Nucl Med Mol Imaging 2007 34 Suppl 2 S229

[B9] Fleming JS Kemp PM A comparison of deconvolution and the Patlak-Rutland plot in renography analysis J Nucl Med 1999 40 1503 1507 10492372

[B10] Trof RJ Di Maggio F Leemreis J Groeneveld AB Biomarkers of acute renal injury and renal failure Shock 2006 26 245 253 10.1097/01.shk.0000225415.5969694.ce 16912649

[B11] Clausen TD Kanstrup IL Iversen J Reference values for 99mTc-MAG3 renography determined in healthy, potential renal donors Clin Physiol Funct Imaging 2002 22 356 60 10.1046/j.1475-097X.2002.00443.x 12487009

[B12] Nogueira JM Weir MR Jacobs S Breault D Klassen D Evans DA Bartlett ST Cooper M A study of renal outcomes in obese living kidney donors Transplantation 2010 90 993 999 20844468 10.1097/TP.0b013e3181f6a058

[B13] Mansoor O Chandar J Rodriguez MM Abitbol CL Seeherunvong W Freundlich M Zilleruelo G Long-term risk of chronic kidney disease in unilateral multicystic dysplastic kidney Pediatr Nephrol 2011 26 597 603 10.1007/s00467-010-1746-0 21240528

[B14] Bomanji J Majeed F Britton KE Nimmon CC Carroll MJ Whitfield HN Radionuclide evaluation pre- and post-extracorporeal shock wave lithotripsy for renal calculi Contrib Nephrol 1987 56 256 60 3301202 10.1159/000413815

[B15] Herrler T Tischer A Meyer A Feiler S Guba M Nowak S Rentsch M Bartenstein P Hacker M Jauch KW The intrinsic renal compartment syndrome: new perspectives in kidney transplantation Transplantation 2010 89 40 46 10.1097/TP.0b013e3181c40aba 20061917

